# Investigations of the Use of Invasive Plant Biomass as an Additive in the Production of Wood-Based Pressed Biofuels, with a Focus on Their Quality and Environmental Impact

**DOI:** 10.3390/plants15020303

**Published:** 2026-01-20

**Authors:** Gvidas Gramauskas, Algirdas Jasinskas, Tomas Vonžodas, Egidijus Lemanas, Kęstutis Venslauskas

**Affiliations:** 1 Department of Agricultural Engineering and Safety, Faculty of Engineering, Agriculture Academy, Vytautas Magnus University, Studentu Str. 15A, Akademija, LT-53362 Kaunas, Lithuania; gvidas.gramauskas@vdu.lt; 2 Thermal Equipment Research and Testing Laboratory, Lithuanian Energy Institute, Breslaujos Str. 3, LT-44403 Kaunas, Lithuania; tomas.vonzodas@lei.lt (T.V.); egidijus.lemanas@lei.lt (E.L.); 3 Department of Mechanical, Energy and Biotechnology Engineering, Faculty of Engineering, Agriculture Academy, Vytautas Magnus University, Studentu Str. 15A, Akademija, LT-53362 Kaunas, Lithuania; kestutis.venslauskas@vdu.lt

**Keywords:** solid biofuel, invasive plants, plant mixtures, pellets, life cycle assessment, combustion, emissions

## Abstract

The present study investigates the suitability of the invasive herbaceous species Sosnowsky’s hogweed (*Heracleum sosnowskyi*) and giant knotweed (*Fallopia sachalinensis*), together with reed (*Phragmites australis*), as feedstock for pressed biofuel pellets used alone and as additives to pinewood. Biomass of the three herbaceous species and pinewood was harvested, dried, chopped, milled, and pelletized through a 6 mm die to obtain pure pellets and binary mixtures of each herbaceous biomass with pinewood (25, 50, and 75% by weight of herbaceous share). The pellets were characterized for physical and mechanical properties, elemental composition, calorific value, combustion emissions, and life cycle impacts per 1 GJ of heat. Pellet density ranged from 1145.60 to 1227.47 kg m^−3^, comparable to or higher than pinewood, while compressive resistance satisfied solid biofuel quality requirements. The lower calorific values of all herbaceous and mixed pellets varied between 16.29 and 17.78 MJ kg^−1^, with increased ash and nitrogen contents at higher herbaceous shares. Combustion tests showed substantially higher CO and NO_x_ emissions for pure invasive and reed pellets than for pinewood, but all values remained within national regulatory limits. Life cycle assessment indicated the highest global warming and fossil fuel depletion potentials for reed systems, followed by Sosnowsky’s hogweed and giant knotweed, with pinewood consistently exhibiting the lowest impacts. Overall, invasive plants and reed are technically suitable as partial pinewood substitutes in pellet production, supporting simultaneous invasive biomass management and renewable heat generation.

## 1. Introduction

Biomass from dedicated and opportunistic energy crops has attracted increasing attention as a sustainable resource for heat and power generation. Biomass is considered a key renewable energy source capable of contributing to climate change mitigation. Compared with solid fossil fuels, plant-based biofuels can substantially reduce net greenhouse gas emissions because the carbon dioxide released during combustion is partially offset by CO_2_ uptake during photosynthesis, resulting in a near-neutral carbon balance over the growth and use cycle [1,2].

The rapid expansion of non-native plant species represents a growing threat to biodiversity, ecosystem functioning, and land use across many regions. Conventional control measures, including mechanical (hoeing, cutting, and digging) and chemical (herbicide-based) approaches, are effective but require considerable financial and labour resources and do not contribute to a circular or low-carbon bioeconomy. Moreover, the use of herbicides may cause unintended impacts on non-target organisms and raise concerns regarding public and occupational health. There is therefore a clear need for cost-effective and environmentally compatible strategies that integrate invasive plant management with value-added biomass utilization [3].

In Lithuania, Sosnowsky’s hogweed (*Heracleum sosnowskyi*) and giant knotweed (*Fallopia sachalinensis*) are among the most widespread invasive herbaceous species, forming dense stands and accumulating substantial above-ground biomass. Reported dry matter yields of Sosnowsky’s hogweed range from 4 to 20 t ha^−1^, while giant knotweed yields are typically 8 to 15 t ha^−1^, depending on site conditions and management [4]. The biomass produced by these species does not require sowing, fertilization, or other agronomic inputs, so the associated cultivation costs are negligible, and the harvested biomass can potentially offset some of the eradication expenses [4]. In addition to invasive species, reed (*Phragmites australis*) forms extensive stands in wetland and littoral zones and represents an abundant herbaceous feedstock that can be mobilized for energy purposes, often in the same landscapes where invasive plants are controlled.

Sosnowsky’s hogweed is a perennial monocarpic species of the *Apiaceae* family, reproducing exclusively by seed and flowering only once before senescence. Each plant can produce approximately 10,000–20,000 seeds, with first-year germination rates reported up to 90%, which underpins the species’ strong competitive ability and rapid spread [5]. The plant’s tissues, particularly the sap, contain photoactive furanocoumarins that are highly toxic; skin contact followed by exposure to solar radiation can cause severe photodermatitis, blistering, and burn-like lesions [6]. These toxic properties necessitate careful handling of biomass during harvesting and processing, but they do not preclude its subsequent energetic use after appropriate safety measures.

Giant knotweed (*Fallopia sachalinensis*), native to eastern Asia, is a tall, rhizomatous perennial capable of reaching heights from 1.5 to over 3.5 m [7]. The species regenerates annually from an extensive rhizome network, enabling rapid recolonization and high productivity, with reported dry matter yields of up to 15 t ha^−1^ yr^−1^ under favourable conditions [8]. Giant knotweed belongs to a complex of closely related taxa, including Japanese knotweed (*Fallopia japonica*), Bohemian knotweed (*Fallopia* × *bohemica*), and Himalayan knotweed (*Persicaria wallichii*), several of which are also recognized as problematic invaders and have been investigated as potential energy crops [7,8].

The sustainable use of herbaceous biomass as solid fuel is regulated through technical standards and quality certification schemes. In Europe, quality requirements for densified biofuels are specified by national and international standard such as DIN 51731 [9] in Germany, while the ENplus certification system, based on CEN/TC, EN, and ISO standards, provides harmonized criteria for wood pellet quality and facilitates market transparency [10]. Compliance with these standards requires pellets to meet threshold values for physical–mechanical properties (e.g., diameter, length, bulk density, and durability), moisture content, ash content, and energy characteristics.

Combustion of herbaceous or mixed biomass is also subject to emission regulations intended to limit air pollutant release. In Lithuania, emission limits for small and medium combustion plants are defined in LAND 43-2013, which specifies maximum permissible concentrations of key pollutants, including nitrogen oxides (NO_x_) (650 mg Nm^−3^), sulphur dioxide (SO_2_), and carbon monoxide (CO) (2000 ppm), and total particulate matter for biofuel-fired installations in the 0.12–1.0 MW thermal output range, standardized to 6% O_2_ in flue gas [11]. Ensuring that pellets produced from invasive and herbaceous biomass comply with these limits is essential for their practical application in small-scale heating systems.

Several studies have highlighted that invasive grasses and tall herbaceous species can serve as additional biofuel sources because these plants must be controlled and removed regardless of energy use [12]. When managed according to sustainable development principles, their utilization for energy can contribute to greenhouse gas mitigation by displacing fossil fuels and reducing open-field decomposition or burning [12,13]. However, the overall environmental performance of such systems is highly sensitive to feedstock characteristics, logistics, densification conditions, and combustion technology [13,14]. To demonstrate the sustainability of invasive biomass utilization pathways and to quantify trade-offs among impact categories, life cycle assessment (LCA) is required alongside conventional fuel quality and emission measurements [13,14,15].

Against this background, the present study investigates the potential of invasive herbaceous biomass from Sosnowsky’s hogweed and giant knotweed, together with reed, for the production of densified solid biofuels. Pure pellets from each herbaceous species and pinewood, as well as binary mixtures of each herbaceous biomass with pinewood at different mass ratios, are produced using a 6 mm die and characterized with respect to physical–mechanical properties, elemental composition, ash and calorific values, and combustion emissions under controlled conditions. In parallel, an attributional LCA is performed using a functional unit (FU) of 1 GJ of useful heat to evaluate the environmental impacts of heat generation from these pellet systems. By combining fuel quality assessment, emission characterization, and LCA, the study aims to determine whether invasive plant biomass and reed can serve as technically and environmentally viable additives or partial substitutes for conventional wood in pressed biofuel production under regional regulatory constraints.

## 2. Materials and Methods

### 2.1. Biomass Pellet Production

#### 2.1.1. Chopping and Milling

The first stage in the biomass processing procedure involves slicing the dried biomass collected from the invasive species giant knotweed, reed, and Sosnowsky’s hogweed, with pinewood as a control. Sosnowskys’s hogweed and giant knotweed plants are fibrous, while reed plants have a texture more like grass. Initially, the biomass is dried and subsequently chopped and milled with specialized equipment to guarantee that the fragments do not exceed 2 mm in diameter. During the chopping and milling, the fibres did not tangle.

#### 2.1.2. Pellet Formation

The raw material, which had been chopped and milled, was pelletized using a biomass granulator with a horizontal matrix with 6 mm diameter and 20 mm length holes, operating at low power (7.5 kW). The granulator had a processing capacity of 100–120 kg h^−1^. Prior to being fed into the granulator, the milled raw material was thoroughly mechanically mixed for homogeneity and lightly moistened with a small quantity of water (0.5–1%). Mixture percentages were measured by weight.

The parameters for the pelleting process were established following the guidelines provided by the manufacturer of ZLSP200B (GEMCO Energy, Anyang, China). A total of 5 kg of prepared material was used, which falls within the specified range. A mill with a moisture content of 12–15% was employed for the production of pellets. The compression ratio, which measures the effective length of the die holes in relation to their diameter, was taken into account. The positions of the roller and die were adjusted according to the manufacturer’s instructions, ensuring that visual control did not exceed 0.3 mm. The temperature within the pellet machine was left unregulated. The length of the pellets was managed by modifying the cutting knife mechanism of the pellet machine to achieve 25–30 mm. After being formed, the pellets were cooled and dried at ambient humidity and temperature.

The biomass pellets originated from the same raw materials. The pellets were made in the Agriculture Academy laboratory at Vytautas Magnus University. The moisture content of the raw material used for pellet creation ranged from 15% to 17%, which was comparable to the moisture level required for pellet production. Furthermore, samples were created by mixing pinewood biomass with biomass of all three invasive plant species. The parameters for the pelleting process were set according to the manufacturer’s specifications. The chopped and ground biomass was fed into the press, where it underwent compression. No binding agents were utilized in the granulation process. During granulation, the biomass was heated to temperatures between 70 and 90 °C. The pellets were subsequently cooled.

### 2.2. Identifying the Key Characteristics of Biomass Pellets

#### 2.2.1. Moisture Content Analysis

Three samples of biomass from each plant were placed in containers and weighed to measure the moisture content of the pellets. Subsequently, the containers, along with the samples, were put in a drying cabinet set at a consistent temperature of 105 °C for a duration of 24 h. Following the drying process, the weight of the dried samples and empty containers was documented. Subsequently, the moisture content of all samples was calculated, including the average and variation according to ISO 18134-3:2023 standards [16].

#### 2.2.2. Pellet Density

To find the density of the pellets, ten pellets were chosen at random and weighed. Then, measurements were taken of the diameter and length of each pellet to calculate their volume and calculate their density. The density of the analysed pellets was determined according to ISO 18847:2024 and ISO 17829:2025 standards [17,18].

#### 2.2.3. Compression Resistance of Pellets

Five pellets from different plants were randomly selected and tested in a lab. A compression testing tool called the INSTRON 5965 with a 5 kN capacity was used. The cylindrical pellets were placed horizontally between two anvils, and compressive force was applied to each pellet. The test was conducted at a compression rate of 20 mm∙min^−1^ and stopped when the pellet fractured. Data were collected using Bluehill tools’ software (version 3), with a data collection interval of 0.1 s.

#### 2.2.4. Pellet Strength Testing

The strength of the produced pellets was evaluated using a testing device. Sample pellets were weighed and then placed into a rectangular container. The electric motor was activated, operating at a frequency of 13 rotations per minute for a duration of three minutes. A receptacle was set beneath the stand to gather the crushed material. Following the 3 min testing interval, the collected mass was weighed, and the difference in weight was calculated to find the percentage of mass that had been lost. Each type of pellet was subjected to this test five times. This method, established by Jasinskas and other researchers, is not standardized and closely resembles the procedure for measuring pellet strength [19].

#### 2.2.5. Statistical Analysis

MS Office Excel was used for the statistical analysis of the results. During data analysis, the suitable number of repetitions, average values, and mean values, along with the 95% confidence intervals of the means, were applied. The least significant difference (LSD05) was determined using a *t*-test at a significance level of *p* ≤ 0.05.

#### 2.2.6. Elemental Composition, Ash Content, and Calorific Value Evaluation

All studies to determine the ash content, elemental composition, and calorific value of the tested samples took place in the Thermal Equipment Testing and Research laboratory at the Lithuanian Energy Institute (LEI). The testing technique is widely accepted in European countries and Lithuania. Three devices, namely, Device Number 8B/5, Device Number 8B/3, and Device Number 8B/2, comply with LST EN ISO 18122:2023 [20] for ash content, LST EN ISO 16948:2015 [21] for elemental composition, and 14918: EN ISO 18125:2017 for calorific value, respectively [22].

#### 2.2.7. Evaluation of Burning Emissions

The LEI laboratory performed emissions tests by burning manufactured pellets in compliance with the LST EN 14785:2006 standard. To measure the quantities of total carbon, nitrogen, sulphur, hydrogen, and oxygen during combustion, combustion product analysers, Datatest 400CEM and VE7, were employed [23].

### 2.3. Life Cycle Assessment

**Figure 1:**
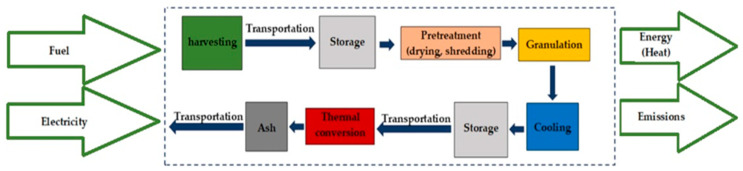
System boundaries: biofuel from invasive plant species usage for energy (authors’ work).

The LCA was conducted in accordance with ISO14040:2006 [24]. The impact assessment was conducted using SimaPro 9.5 software and the CML-I baseline model [25]. Eleven environmental impact categories were considered: global warming (GWP), eutrophication (EP), acidification (AP), ozone layer depletion (ODP), abiotic depletion (AD), photochemical oxidation (PO), terrestrial ecotoxicity (TE), freshwater aquatic ecotoxicity (FWAE), human toxicity (HT), abiotic depletion (fossil fuels) (ADF), and marine aquatic ecotoxicity (MAE). Data on invasive biomass harvesting, transportation, pelleting, and other equipment from the Ecoinvent v3 database were used (see Figure 1) [26].

#### 2.3.1. Functional Unit

As a reference point for comparing LCA results, a functional unit (FU) quantifies a product’s identified functions through inputs and outputs. In this study, the FU is 1 GJ of thermal energy produced by a pellet in a solid fuel boiler.

#### 2.3.2. System Boundary

A full LCA of biomass preparation for biofuel starts with biomass cultivation and ends with ash disposal. The system boundaries considered for this study are shown in Figure 1. They include invasive grass harvesting, transportation of feedstock, pellet production, and combustion in a boiler.

#### 2.3.3. Inventory Analysis

Inventory data quality and reliability were assessed using criteria recommended for LCA studies (temporal, geographical, and technological representativeness; completeness; and methodological consistency). Foreground activity data were prioritized from direct measurements and documented operational parameters; where primary data were unavailable, secondary values were sourced from ecoinvent v3 processes selected to best match the modelled technology level and regional context, and conservative assumptions were applied for uncertain parameters. Cross-checks against literature ranges and internal consistency checks (e.g., energy requirements per functional unit and consistency between fuel ultimate analysis, LCV, and combustion stage inventories) were used to reduce the likelihood of systematic bias and to justify the suitability of the adopted datasets for comparative interpretation.

##### Biomass Harvesting

The biomass of invasive plant species was harvested using a two-step mechanical process involving cutting and baling. Initially, above-ground biomass was mechanically cut using forage harvesters or rotary mowers. The cut material was then collected and compacted into bales using standard agricultural baling equipment. This method enables efficient handling, storage, and transportation of biomass, while minimizing the risk of spreading invasive propagules when combined with appropriate containment measures. Cutting and baling are particularly effective for managing dense stands in accessible terrain and can facilitate subsequent utilization pathways, such as bioenergy production or controlled composting.

##### Transportation

The initial transport of biomass from the harvest site to the intermediate storage facility was modelled over a distance of 3 km, utilizing an agricultural tractor–trailer combination. Subsequent long-distance transport to the granulation plant, thermal conversion plant, and landfill site was assumed to cover 30 km, conducted by a heavy-duty road lorry with EURO 5 emission standards. Data were taken from Ecoinvent v3 [25].

##### Ash Utilization

Following combustion, residual ash was collected from the combustion chamber and filtration systems. It was subsequently transported to a licensed landfill for final disposal, in accordance with local environmental regulations. This practice ensures the safe and compliant management of combustion residues, minimizing potential environmental impacts.

## 3. Results

### 3.1. Qualitative Parameters of Produced Biofuel

#### 3.1.1. Biomass Accumulation and Harvesting

Biomass accumulation patterns of Sosnowsky’s hogweed and giant knotweed revealed distinct growth dynamics (Table 1). Hogweed showed rapid early development, reaching 6.48 t ha^−1^ in May and peaking at 8.96 t ha^−1^ in July before declining to 4.85 t ha^−1^ in August. In contrast, giant knotweed accumulated biomass more gradually, peaking at 6.40 t ha^−1^ in July and maintaining steadier residual growth into August (3.26 t ha^−1^).

These trends reflect species-specific strategies: hogweed invests in rapid growth and seed production, producing higher short-term yields but offering a narrower harvest window, while knotweed sustains biomass accumulation longer, making it more suitable for flexible harvesting regimes [4].

Overall, hogweed achieved higher peak yields, whereas knotweed provided more stable seasonal productivity. Both species, even under conservative estimates, fall within reported productivity ranges—hogweed: 4–20 t ha^−1^; knotweed: 8–15 t ha^−1^ [4].

#### 3.1.2. Physical Properties of Produced Solid Biofuel

The mill utilized biomass with a moisture level of 12–15% to produce pellets. The pellets were formed, and their physical properties, as outlined in Table 2, were analysed. The pellets’ diameter corresponded to that of the granulator matrix, which measured 6 mm. Any increase in the pellets’ diameter occurred due to the expansion of the material when exposed to heat.

Table 2 indicates that the properties of pellets derived from invasive herbaceous plants are quite similar to those obtained from woody pine biomass. The moisture content measured in the analysed pellets was 4.96% for Sosnowky’s hogweed, 7.95% for giant knotweed, 6.82% for reed, and 6.9% for pinewood pellets. There was no significant difference in pellet density between all types of produced pellets, which served as the control. Additionally, there was no notable difference in density between the pinewood pellets and those made from giant knotweed. The pellet density of common reed was the highest, reaching 1341.0 ± 41.94 kg m^−3^.

The researchers Bidhan Nath et al. found that the density of herbaceous plant pellets after adjusting for variance is comparable to that of pinewood pellets [26]. In their study on wheat straw pellets, they observed that the density of the pellets was approximately 1189 kg m^−3^, similar to pinewood pellets. Their research highlighted that the primary distinguishing factor was the length of the pellets.

The pellets made from pinewood biomass were similar in size and weight to the pellets made from invasive herbaceous plants, indicating that the pellets from plants like Sosnowky’s hogweed and giant knotweed are suitable for solid biofuel production (see Table 2).

The researchers Granado et al. have successfully created pellets using cassava rhizome, sugarcane bagasse, and sugarcane straw. These pellets have a density ranging from 1240 to 1300 kg m^−3^, which is greater than that of pellets made from herbaceous plants [27].

After conducting measurements on the briquettes, it was found that the cylindrical pellets had diameters of roughly 6 mm, since the granulation process uses a matrix with holes with a 6 mm diameter. The pellets slightly expanded by 0.1 to 0.2 mm due to a heating process. Based on these findings, it can be concluded that the pellets were uniformly and accurately formed with similar diameters. The measured lengths varied significantly due to differences in the breaking point of the pellets. Another study was conducted where, under similar conditions, the scientists Križan P. et al. produced beech sawdust pellets with a density ranging from 840 kg m^−3^ to 970 kg m^−3^, while analysed invasive herbaceous plants exhibited a greater range from 1145.6 to 1227.5 kg m^−3^ [28].

The researchers Jaya S. Tumuluru and E. Fillerup achieved slightly lower parameters, creating biofuel pellets using a combination of herbaceous and woody biomass. They determined that the pellets had a unit density exceeding 750 kg m^−3^ [29].

The properties of pellets are greatly enhanced in comparison to unprocessed biofuel. The primary benefit is the higher density, leading to improved combustion efficiency and mode adjustment. Additionally, the level of harmful emissions released during combustion is reduced [30] These characteristics fulfil the criteria for the utilization of biofuel.

### 3.2. Solid Biofuel Strength Determination

**Figure 2:**
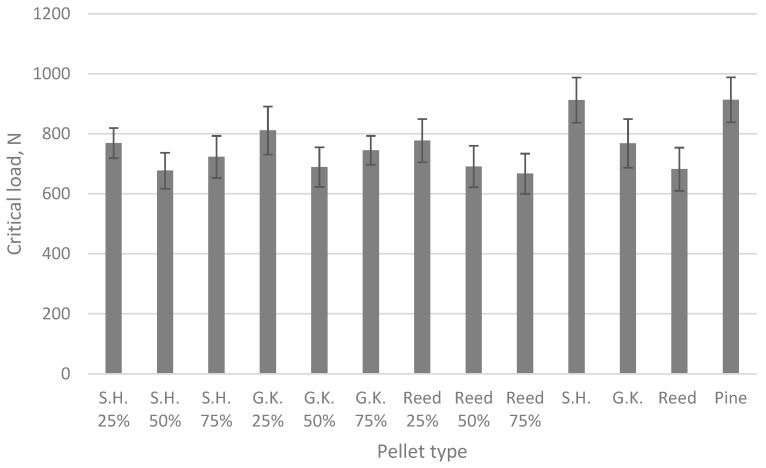
Compressive resistance of pellets (whiskers indicate standard deviations of data).

The strength of pellets made from invasive herbaceous plants, pinewood, and their mixtures was tested to determine their maximum critical loads, as shown in Figure 2. This testing is important to ensure that the pellets can withstand transportation and utilization without being destroyed.

The force–deformation results for pellets made from giant knotweed, Sosnowsky’s hogweed, reed, and pinewood, as well as their mixtures, are displayed in Figure 2. During testing, ten random pellet samples were collected for each type and compared. It can be seen that all pellet samples could withstand loads from about 670 to 920 N, of which the most durable were raw Sosnowsky’s hogweed and pine.

The compression resistance assessment reveals that pellets produced from all three types of herbaceous species fulfil the quality standards set by the European Union.

The densification process quality relies on different factors like moisture content, feed intake speed, particulate size, and biomass quality, so additional research is needed to identify the optimal variables when producing biofuel pellets, according to the researchers Kaliyan Nalladurai and R. Vance Morey [31]. The solid biofuel produced is resilient enough for packaging, transportation, and utilization.

The potential for utilizing fibrous hemp Bialobrzeskie, Futura 75, and Santhica 27 to create compressed biofuel was also investigated by the Latvian researchers Kakitis and colleagues. They studied the pellets’ mechanical properties and strength and determined their critical pressure, which varied from 101.3 to 122.4 N [32].

Nikiforov and his team investigated the fragility of pellets composed of sunflower husks and coal dust mixtures, which exhibited mass losses ranging from 25% to 38%, considerably greater than those of the pellets made from Sosnowsky’s hogweed, giant knotweed, and common reed [33].

### 3.3. Elemental Properties and Emissions of Produced Solid Biofuel

#### 3.3.1. Elemental and Energetic Properties

The elemental analysis presented in Table 3 provides a first-order basis for interpreting combustion emission tendencies in addition to energetic performance. Fuel-bound nitrogen is a principal precursor of NO_x_ in biomass combustion, particularly in small- and medium-scale systems where fuel-N typically dominates over thermal NO_x_ formation; therefore, the elevated nitrogen content of reed (2.02%) and giant knotweed (1.45%) compared with pine (0.83%) indicates an increased NO_x_ formation potential, while Sosnowsky’s hogweed (1.11%) is intermediate. Sulphur contents remained low (<0.05%), suggesting generally limited SO2 formation relative to sulphur-rich fossil fuels; nevertheless, the higher sulphur fraction in reed (0.047%) and giant knotweed (0.033%) compared with pine (0.016%) and Sosnowsky’s hogweed (<0.002%) indicates a comparatively higher SO_2_ emission propensity. Chlorine was below the detection limit (<0.005%) for all investigated fuels, which implies a low risk of HCl-related emissions and chlorine-driven high-temperature corrosion under typical biomass boiler conditions. In parallel, differences in carbon, oxygen, and ash fractions provide mechanistic context for LCV variation: giant knotweed exhibited the highest carbon content and LCV (17.78 MJ kg^−1^), whereas reed had a lower carbon content and the highest ash fraction, which can reduce burnout quality and increase the formation of flak [34].

Researchers across various nations are considering the unique climatic conditions of their regions while exploring new plant types that can be utilized for biofuel, as well as investigating methods for their processing and energy conversion. Latvian researchers, including Adamovics and colleagues, found that the calorific value of fibre hemp cultivated in optimal conditions ranged from 17.8 to 18.98 MJ kg^−1^, with an ash content between 1.5 and 2.7% [35].

Across the investigated pine–invasive biomass blends, ash content and LCV exhibited clear, mixture-dependent trade-offs, as can be seen in Table 4. In the Sosnowsky’s hogweed (S.H.) blends, ash increased markedly with increasing S.H. share (6.16→8.29→10.42%), while LCV decreased (17.32→17.08→16.84 MJ kg^−1^), indicating progressive energetic dilution alongside elevated inorganic residue formation. Reed blends followed the same direction but more strongly: ash rose from 6.96% to 12.82% as reed increased to 75%, accompanied by an LCV decline from 17.24 to 16.61 MJ kg^−1^, representing the least favourable combination of high ash and low LCV among the tested mixtures. In contrast, giant knotweed (G.K.) blends maintained comparatively low ash (4.45–5.31%) while achieving the highest LCV values overall (17.61–17.73 MJ kg^−1^), suggesting that G.K. addition preserved energy density more effectively while limiting ash-related operational burdens.

In addition to the LCV–carbon relationship, the mixture-dependent variation in nitrogen and sulphur contents in Table 4 provides context for the measured gaseous emissions (Table 5). Fuel-bound nitrogen contributes directly to NO_x_ formation; accordingly, fuels and blends with a higher nitrogen content are expected to exhibit higher NO_x_, while it is to be acknowledged that fuel-N-to-NO_x_ conversion is strongly influenced by combustion temperature, excess air, and staging. Sulphur content is directly linked to SO_2_ formation; therefore, the elevated sulphur fraction of reed- and giant knotweed-containing fuels provides a compositional explanation for the higher SO_2_ values observed during combustion. By contrast, elevated CO levels primarily indicate incomplete oxidation (air–fuel mixing and residence-time limitations) rather than ultimate composition alone and thus should be interpreted mainly as combustion quality indicators rather than elemental signatures [36].

The lower calorific value of Sosnowky’s hogweed appears to be comparable to the values in K. Paramonova‘s study, at 16.5 MJ kg^−1^ [37]. This is similar to the calculated lower calorific value of giant knotweed in this study, which was 15.9 ± 1.004 MJ kg^−1^. These findings suggest that the biomass from giant knotweed and Sosnowsky’s hogweed is suitable for solid biofuel production.

#### 3.3.2. Emissions from Solid Biofuel Burning

Research was conducted at the Lithuanian Energy Institute’s laboratory to study the combustion of Sosnowsky’s hogweed and giant knotweed solid biofuel. The emissions from the burning of Sosnowsky’s hogweed, giant knotweed, and reed pressed biofuel were fully measured and are detailed in Table 5. Additionally, solid biofuel manufactured from pinewood was burned to evaluate the discrepancy in emissions.

Solid biofuel produced from invasive herbaceous plants generates higher levels of carbon monoxide compared to biofuel derived from pinewood, but it still adheres to European standards. Utilizing these plants for biofuel helps offset the energy required to eradicate them from the ecosystem. Based on the established criteria, it is evident that reed is better suited for the production of solid biofuel, as can be seen from the data in Table 5. Even though Sosnowsky’s hogweed and giant knotweed biomass yields more volatile end products, it can still serve as an intermediary material for solid biofuel production.

The combustion process was completed entirely. However, according to Table 5, giant knotweed emitted 2667 ppm of carbon monoxide gas due to uneven combustion of the solid biofuel. Sosnowsky’s hogweed also showed variable burning intensity, emitting 1022 ppm of carbon monoxide gas, while reed pellets burned effectively, only emitting 253 ppm. Sosnowky’s hogweed exhibited nitrogen oxide values of 119 ppm, and giant knotweed exhibited values of 249 ppm. All of the values except for that for G.K. (2667 ppm CO), presented in Table 5, fall within the acceptable ranges outlined in the LAND 43-2013 Lithuanian standard for biofuel usage in combustion boilers.

According to other researchers, straw pellets have a carbon monoxide content of 6.4–5.0 g kg^−1^, while wood pellets have a carbon monoxide concentration of 1.3–1.9 g kg^−1^. Nitrogen oxide concentrations were correspondingly 3.608 g kg^−1^ and 1.402 g kg^−1^ [38].

Other researchers calculated that the nitrogen oxide content in peat was 225 mg MJ^−1^, and the carbon monoxide concentration was 140 mg MJ^−1^ [39].

Finnish scientists conducted research on the biofuel created from a combination of wood and reed canary grass, with ratios of 78% and 22%, respectively. The carbon monoxide levels were measured at 963 mg MJ^−1^, and the nitrogen oxide levels were recorded at 212.17 mg MJ^−1^ [40].

Reed canary grass, giant knotweed, and elephant grass quantities of carbon monoxide, nitrogen oxide, and hydrogen chloride were estimated by Czech researchers. Reed canary grass that had been burned was found to release 746 mg m^−3^ of carbon monoxide, 248 mg m^−3^ of nitrogen oxide, and 198 mg m^−3^ of hydrogen chloride. Elephant grass produced 543, 167, and 133 mg m^−3^ of emissions, while giant knotweed produced 249, 300, and 238 mg m^−3^ [41].

Other researchers studied pellets made from giant knotweed. They assessed the levels of carbon monoxide, nitrogen oxide, and hydrogen chloride. The concentrations of three substances were found to be high: carbon monoxide (3088.6 mg m^−3^), nitrogen oxide (194.95 mg m^−3^), and hydrogen chloride (157.69 mg m^−3^). Similar results were observed when wood pellets were burned, with carbon monoxide levels at 2313.6 mg m^−3^, nitrogen oxide levels at 48.5 mg m^−3^, and hydrogen chloride levels at 39.03 mg m^−3^. It is evident that giant knotweed poses a greater risk compared to wood biofuel [42].

### 3.4. Life Cycle Assessment of Invasive Plants

The results show the impacts associated with the generation of 1 GJ of thermal energy supplied by a pellet boiler (Table 6). The results have been presented for the four analysed cases—Sosnowky’s hogweed, giant knotweed, reed, and pinewood.

The results shown in Table 6 indicate that pellets produced from all three herbaceous plants exhibit higher environmental impacts across most categories compared to pinewood. Reed shows the highest values for global warming potential (11.64 kg CO_2_ eq), abiotic depletion of fossil fuels (129.10 MJ), and toxicity-related categories. Sosnowsky’s hogweed and giant knotweed follow a similar pattern but with slightly lower impacts. Pinewood consistently demonstrates the lowest impact. The comparative analysis reveals that invasive herbaceous plants offer potential as alternative biomass resources, although if biomass from invasive herbaceous plants is used as an additive to conventional wood biofuel they would have less environmental impact.

**Figure 3:**
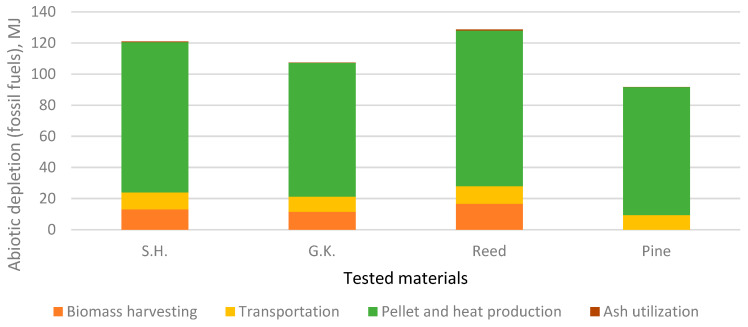
Impact of life cycle stages on abiotic depletion (fossil fuel).

**Figure 4:**
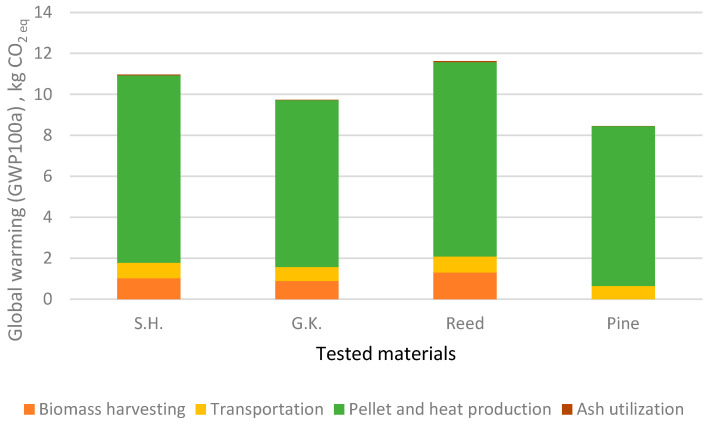
Impact of life cycle stages on global warming (GWP100a).

**Figure 5:**
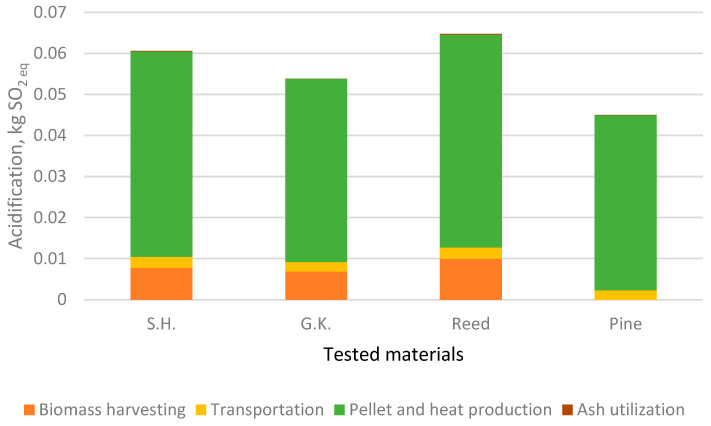
Impact of life cycle stages on acidification.

**Figure 6:**
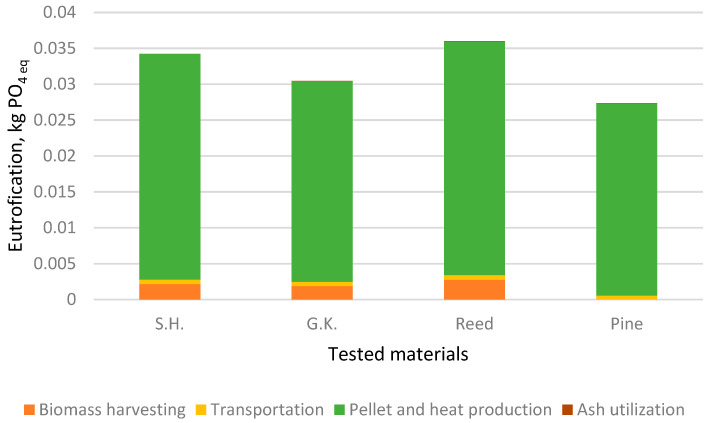
Impact of life cycle stages on eutrophication.

The impact of the different processes of pellet production and use for heat production has been investigated for four impact categories: ADF, GWP, AP, and EP (Figure 3, Figure 4, Figure 5 and Figure 6).

It can be seen that among all four biomass types, pellet and heat production represents the most energy-intensive phase, which is apparent for all four analysed impact categories. Biomass harvesting of the studied invasive herbaceous plants has five times lower impact because there are no costs for cultivation, since invasive plants are self-growing and only need to be harvested. Ash utilization is negligible, since ash from burnt pellets is transported to a landfill.

Based on a comparison of the results with those of scientists such as Petlickaitė et.al. [43], the main benefit of using invasive plant biomass for biofuel production is the reduction in resources that are needed for plant cultivation.

Figure 5 presents the acidification potential (expressed in kg SO_2_ eq) associated with different life cycle stages of pellet production from Sosnowsky’s hogweed (S.H.), giant knotweed (G.K.), reed, and pinewood (Pine). Among the four biomass types, reed exhibits the highest acidification impact (0.064 kg SO_2_ eq), followed by S.H. (0.061 kg SO_2_ eq) and then G.K. (0.054 kg SO_2_ eq), while pinewood shows the lowest value (0.045 kg SO_2_ eq).

In all cases, pellet and heat production is the dominant contributor to acidification, accounting for the majority of emissions. This is primarily due to the release of sulphur and nitrogen oxides during biomass processing and combustion. Minor contributions arise from transportation and biomass harvesting, while ash utilization has a negligible effect. In all cases, pinewood biomass is not directly harvested but sourced from a producer, so its impact on transportation has more variables than compared to other processes.

Reed has the highest impact on ADF, GWP, AP, and EP. This is to be expected since reed biomass has the lowest calorific value and all processes for biofuel production and usage are the same for the analysed plants. Sosnowky’s hogweed and giant knotweed also had a higher impact on all factors than pine. The impact on eutrophication is mostly due to pellet and heat production. Based on a comparison of the results with those of the scientists Želazna et.al. [44], usage of invasive plant biomass for the production of biofuel has an advantage since no resources are used for cultivation.

## 4. Conclusions

This study demonstrated that biomass from Sosnowsky’s hogweed, giant knotweed, and reed, used alone and as additives to pinewood, is technically suitable for pressed solid biofuel production. All herbaceous and mixed pellets exhibited unit densities in the range of 1145.6–1341.0 kg m^−3^, comparable to or higher than pinewood pellets, and compressive resistance of approximately 670–900 N, thereby fulfilling European quality requirements for densified biofuels and ensuring sufficient mechanical durability for handling, transport, and storage. The lower calorific values of pure and mixed pellets were between 16.29 and 17.78 MJ kg^−1^, only slightly lower than those of pinewood, although ash and nitrogen contents increased with higher proportions of herbaceous biomass, particularly for reed.

Combustion and fuel quality results indicate that invasive biomass is most suitable as a partial substitute for wood, and the optimal substitution range depends on plant species when fuel quality (LCV and ash), combustion emissions, and environmental performance are considered together (Table 4, Table 5 and Table 6). For giant knotweed, blends containing 25–50% (by weight) invasive biomass provided the most balanced performance in the tested small-scale boiler, combining low ash (4.45–4.88%) and high LCV (17.61–17.67 MJ kg^−1^) with comparatively low emissions (CO 143–195 ppm; NO_x_ 59–111 ppm); increasing giant knotweed to 75% (by weight) increased CO markedly (1221 ppm), indicating reduced combustion quality under the tested conditions. For Sosnowsky’s hogweed, blends of 25–50% (by weight) were similarly preferred, as ash increased substantially with higher substitution (6.16%→10.42%), and CO increased from 256–402 ppm to 861 ppm at 75% (by weight). For reed, substitution should be limited to ≤25% (by weight) because higher shares substantially increased ash (6.96%→12.82%) and increased SO_2_ emissions 2.33 ppm at 25% (by weight)→5.08–8.52 ppm at 50–75% (by weight), as can be seen in Table 4 and Table 5. Overall, the combined assessment supports 25–50% (by weight) invasive biomass as the recommended substitution window for small-scale combustion applications, with reed restricted to ≤25% (by weight) under the tested conditions.

The life cycle assessment functional unit, 1 GJ of useful heat, indicated higher impacts for invasive herbaceous pellets than for pinewood across the assessed categories, with reed generally exhibiting the highest impacts among the investigated invasive feedstocks (Table 6). When combined with the experimentally observed fuel quality and emission behaviour of blends (Table 4 and Table 5), the LCA results support prioritizing giant knotweed and Sosnowsky’s hogweed over reed for higher substitution levels, and support limiting reed incorporation to low shares. Therefore, an integrative interpretation of pellet properties, combustion emissions, and life cycle results indicates that invasive biomass can be utilized most robustly as a partial wood substitute, with a recommended blend range of 25–50% (by weight) invasive biomass for small-scale heat production, while acknowledging that higher substitution (e.g., up to 75% by weight) may require stricter combustion control and/or emission abatement to avoid elevated CO and ash-related operational expenses.

## Figures and Tables

**Table 1:** Accumulated biomass of invasive plants harvested at the end of each specified month during the year of 2025 (dry mass).

Month	Sosnowsky‘s Hogweed, t ha^−1^	Giant Knotweed, t ha^−1^	Reed, t ha^−1^
April	0.92	1.47	1.11
May	6.48	2.40	3.41
June	7.15	6.15	6.78
July	8.96	6.40	7.26
August	4.85	3.26	4.32

**Table 2:** Physical parameters of produced solid biofuel pellets.

Plant Species	Pellet Parameters				
	Diameter (d), mm	Length (l), mm	Volume (V), m^3^	Mass (m), g	Density (ƍ), kg m^−3^
S.H.	6.2 ± ^1^ 0.22	23.6 ± 0.93	(7.5 ± 0.59) × 10^−7^	0.7 ± 0.08	1145.6 ± 37.50
G.K.	6.1 ± 0.09	23.1 ± 0.84	(6.8 ± 0.25) × 10^−7^	0.7 ± 0.04	1227.5 ± 39.82
Reed	6.1 ± 0.16	22.2 ± 0.68	(5.8 ± 0.23) × 10^−7^	0.6 ± 0.05	1198.3 ± 31.34
S.H. 25% ^2^	6.1 ± 0.20	17.5 ± 0.77	(5.2 ± 0.43) × 10^−7^	0.6 ± 0.06	1124.8 ± 34.16
S.H. 50% ^2^	6.1 ± 0.16	23 ± 0.82	(6.7 ± 0.26) × 10^−7^	0.8 ± 0.02	1188.6 ± 34.82
S.H. 75% ^2^	6.1 ± 0.06	15.9 ± 0.46	(4.7 ± 0.39) × 10^−7^	0.5 ± 0.03	1145.8 ± 42.01
G.K. 25% ^3^	6.1 ± 0.11	21.5 ± 0.97	(6.2 ± 0.34) × 10^−7^	0.8 ± 0.10	1236.5 ± 36.15
G.K. 50% ^3^	6 ± 0.12	17.8 ± 0.58	(5.1 ± 0.55) × 10^−7^	0.7 ± 0.06	1278.0 ± 35.10
G.K. 75% ^3^	6.1 ± 0.21	22.7 ± 0.66	(6.6 ± 0.61) × 10^−7^	0.8 ± 0.03	1205.9 ± 29.86
Reed 25% ^4^	6 ± 0.08	19.5 ± 0.97	(5.5 ± 0.48) × 10^−7^	0.7 ± 0.05	1221.6 ± 32.98
Reed 50% ^4^	6.1 ± 0.19	20.1 ± 0.45	(5.8 ± 0.36) × 10^−7^	0.7 ± 0.08	1154.1 ± 36.76
Reed 75% ^4^	6 ± 0.17	22.2 ± 0.42	(6.3 ± 0.72) × 10^−7^	0.8 ± 0.11	1341.0 ± 41.94
Pinewood	6.1 ± 0.13	30.7 ± 0.88	(8.9 ± 0.83) × 10^−7^	1.1 ± 0.14	1182.5 ± 34.46

^1^ Symbol means the standard deviation of data. ^2^ Sonsnowsky’s hogweed and pine mixture, percentage indicates mass of invasive plant in mixture. ^3^ Giant knotweed and pine mixture, percentage indicates mass of invasive plant in mixture. ^4^ Reed and pine mixture, percentage indicates mass of invasive plant in mixture.

**Table 3:** Elemental properties of invasive plants and pine (control) pellets.

Parameter	S.H.	G.K.	Reed	Pine
Ash, %	12.56 ± ^1^ 0.66	5.74 ± 0.40	15.75 ± 0.05	4.03 ± 0.17
HCV, MJ kg^−1^	17.93 ± 0.60	19.11 ± 0.64	17.62 ± 0.61	18.89 ± 1
LCV, MJ kg^−1^	16.60 ± 0.65	17.78 ± 0.70	16.29 ± 0.67	17.56 ± 1
C, %	46.15 ± 1.87	48.38 ± 1.73	44.79 ± 1.37	49.26 ± 1.22
N, %	1.11 ± 0.31	1.45 ± 0.57	2.02 ± 0.40	0.83 ± 0.39
H, %	5.34 ± 0.67	5.75 ± 0.52	5.14 ± 0.45	5.86 ± 0.67
S, %	<0.002 ^2^	0.033 ± 0.01	0.047 ± 0.01	0.016 ± 0.01
O, %	34.84 ± 0.01	38.65 ± 0.01	32.25 ± 0.01	40.01 ± 0.01
Cl, %	<0.005 ^2^	<0.005 ^2^	<0.005 ^2^	<0.005 ^2^

^1^ Symbol means the standard deviation of data. ^2^ Below detection limit.

**Table 4:** Elemental properties of pellets from invasive plants and pine mixtures.

Parameter	S.H. 25% ^2^	S.H. 50% ^2^	S.H. 75% ^2^	G.K. 25% ^3^	G.K. 50% ^3^	G.K. 75% ^3^	Reed 25% ^4^	Reed 50% ^4^	Reed 75% ^4^
Ash, %	6.16 ± ^1^ 0.32	8.29 ± 0.41	10.42 ± 0.62	4.45 ± 0.39	4.88 ± 0.49	5.31 ± 0.51	6.96 ± 0.22	9.89 ± 0.47	12.82 ± 0.57
HCV, MJ kg^−1^	18.65 ± 0.45	18.41 ± 0.62	18.16 ± 0.27	18.46 ± 0.47	19.00 ± 0.51	19.05 ± 0.39	18.57 ± 0.53	18.25 ± 0.48	17.93 ± 0.47
LCV, MJ kg^−1^	17.32 ± 0.49	17.08 ± 0.57	16.84 ± 0.42	17.61 ± 0.55	17.67 ± 0.39	17.73 ± 0.44	17.24 ± 0.48	16.92 ± 0.39	16.61 ± 0.46
C, %	48.48 ± 1.56	47.70 ± 1.32	46.92 ± 1.22	49.04 ± 1.48	48.38 ± 1.13	48.59 ± 1.61	48.14 ± 1.55	47.06 ± 1.39	45.90 ± 1.68
N, %	0.90 ± 0.23	0.97 ± 0.17	1.04 ± 0.32	0.98 ± 0.22	1.13 ± 0.34	1.29 ± 0.41	1.12 ± 0.30	1.42 ± 0.31	1.72 ± 0.31
H, %	5.72 ± 0.69	5.59 ± 0.66	5.46 ± 0.51	5.83 ± 0.61	5.38 ± 0.78	5.77 ± 0.53	5.67 ± 0.68	5.49 ± 0.62	5.32 ± 0.48
S, %	0.012 ± 0.01	0.301 ± 0.01	0.005 ± 0.01	0.02 ± 0.01	0.302 ± 0.01	0.02 ± 0.01	0.02 ± 0.01	0.03 ± 0.01	0.03 ± 0.01
O, %	38.71 ± 0.01	37.34 ± 0.01	36.12 ± 0.01	39.66 ± 0.01	33.32 ± 0.01	38.98 ± 0.01	38.06 ± 0.01	36.12 ± 0.01	34.18 ± 0.01
Cl, %	<0.005 ^5^	<0.005 ^5^	<0.005 ^5^	<0.005 ^5^	<0.005 ^5^	<0.005 ^5^	<0.005 ^5^	<0.005 ^5^	<0.005 ^5^

^1^ Symbol means the standard deviation of data. ^2^ Sonsnowsky’s hogweed and pine mixture, percentage indicates mass of invasive plant in mixture. ^3^ Giant knotweed and pine mixture, percentage indicates mass of invasive plant in mixture. ^4^ Reed and pine mixture, percentage indicates mass of invasive plant in mixture. ^5^ Below detection limit.

**Table 5:** Emissions from produced biofuel pellets.

Plant Species	H_2_O, %	CO_2_, %	O_2_, %	CO, ppm	NO_x_, ppm	C_x_H_y_, ppm	SO_2_, ppm
S.H.	3.71	3.96	15.1	1022	119	29	0.17
G.K.	4.05	4.21	14.3	2667	249	164	3.68
Reed	3.56	4.03	15.0	253	174	16	4.16
S.H. 25% + pine 75%	4.07	4.54	14.4	256	79	13	0.83
S.H. 50% + pine 50%	3.79	4.28	14.8	402	96	18	0.21
S.H. 75% + pine 25%	3.84	3.89	15.1	861	105	36	0.72
G.K. 25% + pine 75%	3.91	4.15	14.7	143	59	13	1.91
G.K. 50% + pine 50%	4.22	4.51	14.4	195	111	14	1.08
G.K. 75% + pine 25%	3.93	4.12	14.6	1221	131	47	1.45
Reed 25% + pine 75%	3.96	4.31	14.7	174	113	18	2.33
Reed 50% + pine 50%	3.44	3.71	15.5	285	123	25	5.08
Reed 75% + pine 25%	3.83	4.02	15.0	232	152	18	8.52
Pinewood pellets	3.81	4.33	14.7	125	59	9	0.01

**Table 6:** Characterized impacts associated with the pellet chain per functional unit (FU).

Impact Category	Unit	Sosnowky’s Hogweed	Giant Knotweed	Reed	Pine
AD	kg Sb_eq_	1.19 × 10^−3^	1.06 × 10^−3^	1.24 × 10^−3^	1.01 × 10^−3^
ADF	MJ	121.31	107.69	129.10	91.99
GWP	kg CO_2eq_	10.98	9.76	11.64	8.47
ODT	kg CFC-11_eq_	9.12 × 10^−7^	8.08 × 10^−7^	9.83 × 10^−7^	6.49 × 10^−7^
HT	kg 1,4-DB_eq_	56.36	50.18	58.61	47.33
FWAE	kg 1,4-DB_eq_	35.94	32.03	37.98	30.16
MAE	kg 1,4-DB_eq_	50,676.49	45,128.05	52,724.16	42,436.43
TE	kg 1,4-DB_eq_	6.08 × 10^−2^	5.42 × 10^−2^	6.33 × 10^−2^	5.08 × 10^−2^
PO	kg C_2_H_4eq_	3.72 × 10^−3^	3.32 × 10^−3^	3.93 × 10^−3^	2.94 × 10^−3^
AP	kg SO_2eq_	6.07 × 10^−2^	5.39 × 10^−2^	6.48 × 10^−2^	4.50 × 10^−2^
EP	kg PO_4eq_	3.43 × 10^−2^	3.05 × 10^−2^	3.61 × 10^−2^	2.73 × 10^−2^

## Data Availability

The original contributions presented in this study are included in the article. Further inquiries can be directed to the corresponding author.

## Abbreviations

The following abbreviations are used in this manuscript:

S.H.	Sosnowky’s hogweed
G.K.	Giant knotweed
FU	Functional unit
AD	Abiotic depletion
ADF	Abiotic depletion (fossil fuels)
GWP	Global warming (GWP100a)
ODT	Ozone layer depletion (ODP)
HT	Human toxicity
FWAE	Fresh water aquatic ecotoxicity
MAE	Marine aquatic ecotoxicity
TE	Terrestrial ecotoxicity
PO	Photochemical oxidation
AP	Acidification
EP	Eutrophication
HCV	Higher calorific value
LCV	Lower calorific value
